# Dichloridodi-μ_2_-hydroxido-di-μ_3_-oxido-octa­phenyl­tetra­tin(IV) dimethyl sulfoxide disolvate

**DOI:** 10.1107/S1600536812051744

**Published:** 2013-01-09

**Authors:** Shahrbano Foladi, Parivash Khazaei, Jafar Attar Gharamaleki, Behrouz Notash, Mohammad Kazem Rofouei

**Affiliations:** a Department of Chemistry, Faculty of Science, Karaj Branch, Islamic Azad, University, Karaj, Iran; b Faculty of Chemistry, Tarbiat Moallem University, Tehran, Iran; c Department of Chemistry, Shahid Beheshti University, G. C., Evin, Tehran, 1983963113, Iran

## Abstract

In the centrosymmetric tetra­nuclear title mol­ecule, [Sn_4_(C_6_H_5_)_8_Cl_2_O_2_(OH)_2_]·2C_2_H_6_OS, the two independent tin^IV^ atoms show distorted trigonal–bipyramidal SnC_2_O_3_ and SnC_2_O_2_Cl coordination geometries. The four tin^IV^ atoms are bridged by the hydroxo and oxo ligands, forming a ladder-like array of three edge-connected Sn_2_O_2_ squares. The solvent mol­ecules are linked to the tetra­nuclear mol­ecule *via* O–H⋯O hydrogen bonds.

## Related literature

For biological applications of organotin(IV) complexes, see: Davies & Smith (1982 ▶). For the crystal structures of closely related compounds, see: Vollano *et al.* (1984 ▶); Kresinski *et al.* (1994 ▶); Yap *et al.* (2010 ▶).
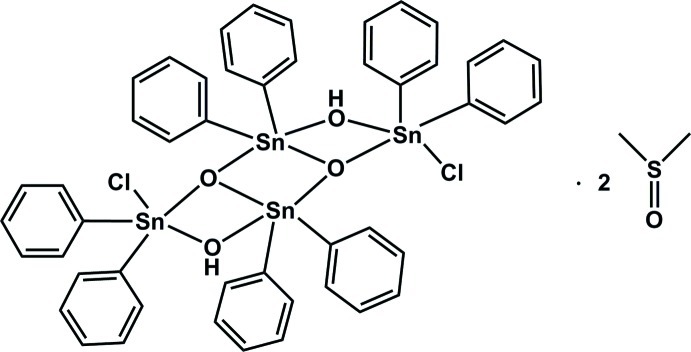


## Experimental

### 

#### Crystal data

[Sn_4_(C_6_H_5_)_8_Cl_2_O_2_(OH)_2_]·2C_2_H_6_OS
*M* *_r_* = 1384.73
Monoclinic, 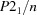

*a* = 11.521 (2) Å
*b* = 19.372 (4) Å
*c* = 11.854 (2) Å
β = 93.61 (3)°
*V* = 2640.4 (9) Å^3^
*Z* = 2
Mo *K*α radiation
μ = 2.10 mm^−1^
*T* = 298 K
0.50 × 0.47 × 0.45 mm

#### Data collection

STOE IPDS 2T diffractometer
Absorption correction: numerical (*X-RED32*; Stoe & Cie, 2005 ▶) *T*_min_ = 0.420, *T*_max_ = 0.452
28831 measured reflections
7108 independent reflections
6034 reflections with *I* > 2σ(*I*)
*R*_int_ = 0.095

#### Refinement

*R*[*F* ^2^ > 2σ(*F* ^2^)] = 0.030
*wR*(*F* ^2^) = 0.079
*S* = 1.05
7108 reflections
304 parameters
1 restraint
H atoms treated by a mixture of independent and constrained refinement
Δρ_max_ = 0.92 e Å^−3^
Δρ_min_ = −0.83 e Å^−3^

### 

Data collection: *X-AREA* (Stoe & Cie, 2005 ▶); cell refinement: *X-AREA*; data reduction: *X-AREA*; program(s) used to solve structure: *SHELXS97* (Sheldrick, 2008 ▶); program(s) used to refine structure: *SHELXL97* (Sheldrick, 2008 ▶); molecular graphics: *ORTEP-3 for Windows* (Farrugia, 2012 ▶); software used to prepare material for publication: *WinGX* (Farrugia, 2012 ▶).

## Supplementary Material

Crystal structure: contains datablock(s) I, global. DOI: 10.1107/S1600536812051744/cv5369sup1.cif

Structure factors: contains datablock(s) I. DOI: 10.1107/S1600536812051744/cv5369Isup2.hkl

Additional supplementary materials:  crystallographic information; 3D view; checkCIF report

## Figures and Tables

**Table 1:** Hydrogen-bond geometry (Å, °)

*D*—H⋯*A*	*D*—H	H⋯*A*	*D*⋯*A*	*D*—H⋯*A*
O2—H2*A*⋯O3^i^	0.82 (2)	2.04 (2)	2.851 (3)	166 (4)

Symmetry code: (i) 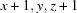.

**Fig. 1.**
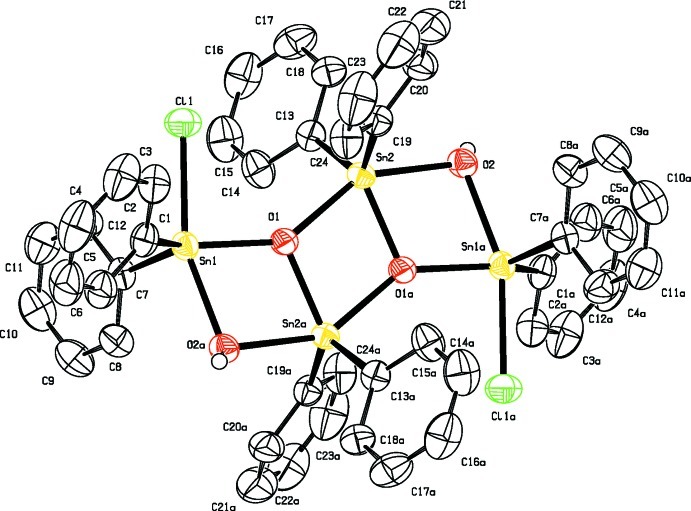
View of (I) showing the atomic numbering and 50% probability displacement ellipsoids [symmetry code: (a) 2-x, -y, 2-z]. Solvent molecule and C-bound H atoms were omitted for clarity.

## supplementary crystallographic information

### Comment

Organotin(IV) complexes have been studied due to the diversity of structures that such compounds can form and in view of their potential biological activities (Davies & Smith, 1982). Herewith we present the title compound (I).

In (I) (Fig. 1), all geometric parameters are normal and correspond to those reported for related compounds (Vollano *et al.*, 1984; Kresinski *et al.*, 1994) The similar structure with bromide (instead of chloride) anions was reported by Yap *et al.* (2010). All tin atoms are five-coordinated, form distorted trigonal–bipyramidal environments. Oxide or hydroxide groups play bridging role between Sn atoms. Each of the inner Sn^IV^ atoms is coordinated by three O atoms in the equatorial plane and two phenyl rings in axial position. The equatorial angle is shorter than ideally 180° being only 124.24 (11) °. The Sn2—O1 and Sn2—O2 bond distances are 2.0451 (18) and 2.1630 (19) Å, respectively. Each of the outer Sn^IV^ atoms is coordinated by one chloride and two O atoms in equatorial plane and axial positions are occupied by two phenyl rings. The Sn1—Cl1 bond distance is 2.4628 (9) Å and axial angle, C1—Sn2—C7 is 120.35 (11) °. The centrosymmetric tetrameric species bears a central part which consists of Sn_2_O_2_ ring with two adjacent Sn_2_O(OH) four-membered rings. This behavior is also consistent with the reported structure. The DMSO molecules accompany the tetranuclear compound by O2—H2A···O3 hydrogen bonds.

### Experimental

The solution of 2-mercaptobenzaldehyde (2.76 g, 20 mmol) in 15 ml e thanol was added to solution of diethylamine (0.6 g, 10 mmol) in 10 ml e thanol. The obtained mixture was refluxed at 60 C for 4 h. The yellow crystals of the product was filtered off and dried. In order to synthesis of the title compound, the obtained ligand and dichloridediphenyltin were dissolved in DMSO at ambient temperature. Colourless crystals of the tetramer suitable for X-ray were obtained by slow evaporation of the solvent within one month.

### Refinement

O-bound H atom was found in a difference Fourier map and isotropically refined with O–H distance restraint of 0.824 (19) Å. C-bound H atoms were positioned geometrically and refined as riding atoms with C—H = 0.93-0.96 Å, and *U*_iso_(H) = 1.2-1.5 *U*_eq_(C).

### Crystal data

**Table:**

[Sn_4_(C_6_H_5_)_8_Cl_2_O_2_(OH)_2_]·2C_2_H_6_OS	*F*(000) = 1360
*M**_r_* = 1384.73	*D*_x_ = 1.742 Mg m^−^^3^
Monoclinic, *P*2_1_/*n*	Mo *K*α radiation, λ = 0.71073 Å
Hall symbol: -P 2yn	Cell parameters from 7108 reflections
*a* = 11.521 (2) Å	θ = 2.0–29.2°
*b* = 19.372 (4) Å	µ = 2.10 mm^−^^1^
*c* = 11.854 (2) Å	*T* = 298 K
β = 93.61 (3)°	Block, colourless
*V* = 2640.4 (9) Å^3^	0.50 × 0.47 × 0.45 mm
*Z* = 2	

### Data collection

**Table:**

STOE IPDS 2T diffractometer	7108 independent reflections
Radiation source: fine-focus sealed tube	6034 reflections with *I* > 2σ(*I*)
Graphite monochromator	*R*_int_ = 0.095
Detector resolution: 0.15 pixels mm^-1^	θ_max_ = 29.2°, θ_min_ = 2.0°
rotation method scans	*h* = −15→14
Absorption correction: numerical (*X-RED32*; Stoe & Cie, 2005)	*k* = −26→26
*T*_min_ = 0.420, *T*_max_ = 0.452	*l* = −16→16
28831 measured reflections	

### Refinement

**Table:**

Refinement on *F*^2^	Primary atom site location: structure-invariant direct methods
Least-squares matrix: full	Secondary atom site location: difference Fourier map
*R*[*F*^2^ > 2σ(*F*^2^)] = 0.030	Hydrogen site location: inferred from neighbouring sites
*wR*(*F*^2^) = 0.079	H atoms treated by a mixture of independent and constrained refinement
*S* = 1.05	*w* = 1/[σ^2^(*F*_o_^2^) + (0.0328*P*)^2^ + 0.0145*P*] where *P* = (*F*_o_^2^ + 2*F*_c_^2^)/3
7108 reflections	(Δ/σ)_max_ = 0.002
304 parameters	Δρ_max_ = 0.92 e Å^−^^3^
1 restraint	Δρ_min_ = −0.83 e Å^−^^3^

### Special details

**Table:**

Geometry. All e.s.d.'s (except the e.s.d. in the dihedral angle between two l.s. planes) are estimated using the full covariance matrix. The cell e.s.d.'s are taken into account individually in the estimation of e.s.d.'s in distances, angles and torsion angles; correlations between e.s.d.'s in cell parameters are only used when they are defined by crystal symmetry. An approximate (isotropic) treatment of cell e.s.d.'s is used for estimating e.s.d.'s involving l.s. planes.
Refinement. Refinement of *F*^2^ against ALL reflections. The weighted *R*-factor *wR* and goodness of fit *S* are based on *F*^2^, conventional *R*-factors *R* are based on *F*, with *F* set to zero for negative *F*^2^. The threshold expression of *F*^2^ > σ(*F*^2^) is used only for calculating *R*-factors(gt) *etc*. and is not relevant to the choice of reflections for refinement. *R*-factors based on *F*^2^ are statistically about twice as large as those based on *F*, and *R*- factors based on ALL data will be even larger.

### Fractional atomic coordinates and isotropic or equivalent isotropic displacement parameters (Å^2^)

**Table:**

	*x*	*y*	*z*	*U*_iso_*/*U*_eq_	
Sn1	0.923073 (16)	0.109727 (9)	0.801260 (13)	0.03225 (6)	
Sn2	1.050659 (15)	0.052478 (9)	1.103601 (13)	0.02920 (5)	
Cl1	0.98076 (8)	0.20923 (4)	0.92137 (6)	0.05121 (18)	
S1	0.46511 (8)	0.04222 (5)	0.28731 (8)	0.0587 (2)	
O1	0.96963 (18)	0.04965 (9)	0.93664 (14)	0.0343 (4)	
O2	1.12468 (17)	−0.00375 (10)	1.24848 (15)	0.0344 (4)	
O3	0.3549 (2)	0.02385 (16)	0.3395 (2)	0.0620 (6)	
C1	0.7530 (3)	0.14934 (15)	0.7610 (2)	0.0386 (6)	
C2	0.6974 (3)	0.19241 (18)	0.8329 (3)	0.0491 (7)	
H2	0.7309	0.2002	0.9052	0.059*	
C3	0.5946 (4)	0.2238 (2)	0.8011 (4)	0.0655 (11)	
H3	0.5579	0.2518	0.8517	0.079*	
C4	0.5453 (3)	0.2137 (2)	0.6929 (4)	0.0697 (11)	
H4	0.4764	0.2360	0.6697	0.084*	
C5	0.5987 (4)	0.1705 (2)	0.6196 (3)	0.0676 (11)	
H5	0.5652	0.1630	0.5472	0.081*	
C6	0.7027 (3)	0.13809 (19)	0.6540 (3)	0.0508 (7)	
H6	0.7383	0.1087	0.6046	0.061*	
C7	1.0545 (3)	0.11797 (15)	0.6825 (2)	0.0376 (6)	
C8	1.0729 (3)	0.06512 (18)	0.6064 (2)	0.0463 (7)	
H8	1.0281	0.0253	0.6075	0.056*	
C9	1.1578 (3)	0.0713 (2)	0.5289 (3)	0.0558 (8)	
H9	1.1704	0.0354	0.4790	0.067*	
C10	1.2236 (3)	0.1309 (2)	0.5260 (3)	0.0581 (9)	
H10	1.2792	0.1357	0.4729	0.070*	
C11	1.2069 (3)	0.1829 (2)	0.6012 (3)	0.0605 (9)	
H11	1.2520	0.2227	0.5999	0.073*	
C12	1.1228 (3)	0.17680 (18)	0.6797 (3)	0.0510 (8)	
H12	1.1124	0.2124	0.7308	0.061*	
C13	1.2033 (2)	0.10738 (14)	1.0699 (2)	0.0343 (5)	
C14	1.2472 (3)	0.10957 (18)	0.9635 (3)	0.0491 (7)	
H14	1.2115	0.0842	0.9045	0.059*	
C15	1.3439 (3)	0.1494 (2)	0.9450 (3)	0.0626 (10)	
H15	1.3732	0.1506	0.8736	0.075*	
C16	1.3969 (3)	0.1874 (2)	1.0319 (4)	0.0640 (10)	
H16	1.4614	0.2144	1.0186	0.077*	
C17	1.3556 (3)	0.18588 (19)	1.1379 (3)	0.0585 (9)	
H17	1.3923	0.2112	1.1965	0.070*	
C18	1.2580 (3)	0.14597 (16)	1.1566 (3)	0.0431 (6)	
H18	1.2290	0.1452	1.2281	0.052*	
C19	0.9102 (2)	0.09486 (14)	1.1881 (2)	0.0356 (5)	
C20	0.9278 (3)	0.11474 (17)	1.3006 (2)	0.0489 (8)	
H20	0.9998	0.1075	1.3388	0.059*	
C21	0.8387 (4)	0.1453 (2)	1.3562 (3)	0.0680 (12)	
H21	0.8512	0.1586	1.4313	0.082*	
C22	0.7322 (4)	0.1558 (2)	1.3006 (4)	0.0762 (14)	
H22	0.6725	0.1759	1.3384	0.091*	
C23	0.7139 (4)	0.1373 (2)	1.1918 (4)	0.0757 (13)	
H23	0.6415	0.1450	1.1548	0.091*	
C24	0.8021 (3)	0.10635 (18)	1.1330 (3)	0.0501 (8)	
H24	0.7883	0.0937	1.0577	0.060*	
C25	0.4630 (5)	−0.0023 (3)	0.1581 (4)	0.0878 (16)	
H25A	0.4487	−0.0504	0.1708	0.132*	
H25B	0.5366	0.0031	0.1255	0.132*	
H25C	0.4024	0.0161	0.1074	0.132*	
C26	0.5777 (4)	−0.0069 (3)	0.3584 (4)	0.0856 (15)	
H26A	0.5851	0.0063	0.4366	0.128*	
H26B	0.6498	0.0017	0.3243	0.128*	
H26C	0.5590	−0.0551	0.3526	0.128*	
H2A	1.1948 (17)	0.000 (2)	1.265 (3)	0.056 (11)*	

### Atomic displacement parameters (Å^2^)

**Table:**

	*U*^11^	*U*^22^	*U*^33^	*U*^12^	*U*^13^	*U*^23^
Sn1	0.03346 (10)	0.03404 (10)	0.02899 (9)	0.00335 (7)	0.00002 (7)	0.00140 (6)
Sn2	0.02770 (9)	0.03127 (9)	0.02838 (9)	0.00062 (6)	−0.00011 (6)	−0.00281 (6)
Cl1	0.0625 (5)	0.0413 (4)	0.0486 (4)	0.0008 (3)	−0.0060 (3)	−0.0099 (3)
S1	0.0438 (5)	0.0623 (6)	0.0697 (5)	0.0040 (4)	0.0006 (4)	0.0115 (4)
O1	0.0412 (11)	0.0300 (9)	0.0305 (8)	0.0013 (8)	−0.0078 (7)	−0.0007 (6)
O2	0.0310 (10)	0.0380 (10)	0.0335 (8)	0.0001 (8)	−0.0050 (7)	−0.0015 (7)
O3	0.0473 (14)	0.0759 (18)	0.0629 (14)	0.0007 (13)	0.0047 (11)	−0.0011 (13)
C1	0.0339 (14)	0.0383 (14)	0.0435 (14)	0.0037 (11)	0.0002 (11)	0.0092 (11)
C2	0.0469 (18)	0.0511 (18)	0.0506 (16)	0.0107 (14)	0.0125 (14)	0.0096 (13)
C3	0.056 (2)	0.058 (2)	0.086 (3)	0.0166 (18)	0.026 (2)	0.0201 (19)
C4	0.0402 (18)	0.068 (3)	0.100 (3)	0.0114 (18)	−0.0023 (19)	0.034 (2)
C5	0.061 (2)	0.072 (3)	0.067 (2)	0.004 (2)	−0.0175 (19)	0.0212 (19)
C6	0.0478 (18)	0.0527 (18)	0.0509 (17)	0.0036 (15)	−0.0049 (14)	0.0092 (14)
C7	0.0392 (15)	0.0417 (15)	0.0318 (12)	0.0018 (12)	0.0014 (11)	0.0048 (10)
C8	0.0459 (17)	0.0531 (18)	0.0400 (14)	−0.0018 (14)	0.0040 (13)	−0.0037 (12)
C9	0.050 (2)	0.076 (2)	0.0417 (15)	0.0036 (18)	0.0085 (14)	−0.0083 (15)
C10	0.0400 (17)	0.087 (3)	0.0482 (17)	0.0027 (18)	0.0115 (14)	0.0055 (17)
C11	0.053 (2)	0.060 (2)	0.069 (2)	−0.0105 (17)	0.0147 (18)	0.0113 (17)
C12	0.054 (2)	0.0446 (17)	0.0554 (17)	−0.0063 (14)	0.0116 (15)	−0.0018 (13)
C13	0.0267 (12)	0.0376 (14)	0.0383 (13)	0.0003 (10)	−0.0010 (10)	0.0024 (10)
C14	0.0414 (17)	0.065 (2)	0.0411 (15)	−0.0018 (15)	0.0037 (13)	0.0044 (13)
C15	0.046 (2)	0.081 (3)	0.062 (2)	−0.0063 (19)	0.0154 (17)	0.0149 (18)
C16	0.0386 (18)	0.066 (2)	0.087 (3)	−0.0126 (17)	−0.0001 (18)	0.021 (2)
C17	0.0457 (19)	0.0465 (19)	0.082 (2)	−0.0079 (15)	−0.0082 (17)	−0.0068 (16)
C18	0.0403 (16)	0.0405 (15)	0.0483 (15)	−0.0018 (12)	0.0012 (12)	−0.0045 (12)
C19	0.0350 (14)	0.0334 (13)	0.0389 (13)	0.0036 (11)	0.0073 (11)	0.0028 (10)
C20	0.057 (2)	0.0464 (17)	0.0440 (16)	−0.0026 (14)	0.0115 (15)	−0.0073 (12)
C21	0.091 (3)	0.054 (2)	0.064 (2)	0.003 (2)	0.039 (2)	−0.0101 (16)
C22	0.076 (3)	0.059 (2)	0.099 (3)	0.020 (2)	0.053 (3)	0.010 (2)
C23	0.050 (2)	0.077 (3)	0.103 (3)	0.028 (2)	0.026 (2)	0.031 (2)
C24	0.0365 (16)	0.060 (2)	0.0539 (17)	0.0081 (14)	0.0049 (13)	0.0122 (14)
C25	0.074 (3)	0.123 (4)	0.067 (2)	0.034 (3)	0.013 (2)	0.002 (3)
C26	0.050 (2)	0.105 (4)	0.099 (3)	0.007 (2)	−0.014 (2)	0.033 (3)

### Geometric parameters (Å, º)

**Table:**

Sn1—O1	2.0271 (18)	C10—C11	1.367 (6)
Sn1—C1	2.130 (3)	C10—H10	0.9300
Sn1—C7	2.137 (3)	C11—C12	1.389 (5)
Sn1—O2^i^	2.196 (2)	C11—H11	0.9300
Sn1—Cl1	2.4628 (9)	C12—H12	0.9300
Sn2—O1^i^	2.0451 (18)	C13—C14	1.388 (4)
Sn2—C13	2.115 (3)	C13—C18	1.390 (4)
Sn2—C19	2.121 (3)	C14—C15	1.385 (5)
Sn2—O1	2.1351 (18)	C14—H14	0.9300
Sn2—O2	2.1630 (19)	C15—C16	1.377 (6)
S1—O3	1.490 (3)	C15—H15	0.9300
S1—C25	1.757 (5)	C16—C17	1.371 (5)
S1—C26	1.777 (4)	C16—H16	0.9300
O1—Sn2^i^	2.0451 (18)	C17—C18	1.394 (5)
O2—Sn1^i^	2.196 (2)	C17—H17	0.9300
O2—H2A	0.824 (19)	C18—H18	0.9300
C1—C2	1.379 (4)	C19—C24	1.387 (4)
C1—C6	1.379 (4)	C19—C20	1.390 (4)
C2—C3	1.363 (5)	C20—C21	1.387 (5)
C2—H2	0.9300	C20—H20	0.9300
C3—C4	1.384 (6)	C21—C22	1.370 (7)
C3—H3	0.9300	C21—H21	0.9300
C4—C5	1.379 (6)	C22—C23	1.343 (7)
C4—H4	0.9300	C22—H22	0.9300
C5—C6	1.390 (5)	C23—C24	1.402 (5)
C5—H5	0.9300	C23—H23	0.9300
C6—H6	0.9300	C24—H24	0.9300
C7—C12	1.387 (4)	C25—H25A	0.9600
C7—C8	1.389 (4)	C25—H25B	0.9600
C8—C9	1.389 (4)	C25—H25C	0.9600
C8—H8	0.9300	C26—H26A	0.9600
C9—C10	1.384 (6)	C26—H26B	0.9600
C9—H9	0.9300	C26—H26C	0.9600
			
O1—Sn1—C1	125.30 (10)	C11—C10—H10	120.0
O1—Sn1—C7	113.66 (10)	C9—C10—H10	120.0
C1—Sn1—C7	120.35 (11)	C10—C11—C12	120.5 (4)
O1—Sn1—O2^i^	74.05 (7)	C10—C11—H11	119.8
C1—Sn1—O2^i^	93.75 (10)	C12—C11—H11	119.8
C7—Sn1—O2^i^	93.94 (9)	C7—C12—C11	120.4 (3)
O1—Sn1—Cl1	86.75 (6)	C7—C12—H12	119.8
C1—Sn1—Cl1	93.34 (9)	C11—C12—H12	119.8
C7—Sn1—Cl1	98.39 (8)	C14—C13—C18	118.8 (3)
O2^i^—Sn1—Cl1	160.17 (5)	C14—C13—Sn2	122.8 (2)
O1^i^—Sn2—C13	121.78 (9)	C18—C13—Sn2	118.3 (2)
O1^i^—Sn2—C19	113.91 (10)	C15—C14—C13	120.3 (3)
C13—Sn2—C19	124.24 (11)	C15—C14—H14	119.9
O1^i^—Sn2—O1	73.87 (8)	C13—C14—H14	119.9
C13—Sn2—O1	99.07 (9)	C16—C15—C14	120.1 (3)
C19—Sn2—O1	98.37 (10)	C16—C15—H15	119.9
O1^i^—Sn2—O2	74.43 (7)	C14—C15—H15	119.9
C13—Sn2—O2	96.46 (9)	C17—C16—C15	120.7 (3)
C19—Sn2—O2	95.42 (9)	C17—C16—H16	119.6
O1—Sn2—O2	148.27 (7)	C15—C16—H16	119.6
O3—S1—C25	106.2 (2)	C16—C17—C18	119.2 (3)
O3—S1—C26	106.9 (2)	C16—C17—H17	120.4
C25—S1—C26	96.9 (3)	C18—C17—H17	120.4
Sn1—O1—Sn2^i^	110.49 (8)	C13—C18—C17	120.8 (3)
Sn1—O1—Sn2	142.90 (9)	C13—C18—H18	119.6
Sn2^i^—O1—Sn2	106.13 (8)	C17—C18—H18	119.6
Sn2—O2—Sn1^i^	100.26 (8)	C24—C19—C20	118.6 (3)
Sn2—O2—H2A	118 (3)	C24—C19—Sn2	121.9 (2)
Sn1^i^—O2—H2A	112 (3)	C20—C19—Sn2	119.4 (2)
C2—C1—C6	118.6 (3)	C21—C20—C19	120.4 (4)
C2—C1—Sn1	122.4 (2)	C21—C20—H20	119.8
C6—C1—Sn1	118.5 (2)	C19—C20—H20	119.8
C3—C2—C1	121.9 (4)	C22—C21—C20	120.1 (4)
C3—C2—H2	119.0	C22—C21—H21	119.9
C1—C2—H2	119.0	C20—C21—H21	119.9
C2—C3—C4	119.5 (4)	C23—C22—C21	120.2 (3)
C2—C3—H3	120.3	C23—C22—H22	119.9
C4—C3—H3	120.3	C21—C22—H22	119.9
C5—C4—C3	119.8 (3)	C22—C23—C24	121.1 (4)
C5—C4—H4	120.1	C22—C23—H23	119.5
C3—C4—H4	120.1	C24—C23—H23	119.5
C4—C5—C6	120.0 (4)	C19—C24—C23	119.5 (4)
C4—C5—H5	120.0	C19—C24—H24	120.2
C6—C5—H5	120.0	C23—C24—H24	120.2
C1—C6—C5	120.2 (4)	S1—C25—H25A	109.5
C1—C6—H6	119.9	S1—C25—H25B	109.5
C5—C6—H6	119.9	H25A—C25—H25B	109.5
C12—C7—C8	118.7 (3)	S1—C25—H25C	109.5
C12—C7—Sn1	120.2 (2)	H25A—C25—H25C	109.5
C8—C7—Sn1	121.1 (2)	H25B—C25—H25C	109.5
C9—C8—C7	120.6 (3)	S1—C26—H26A	109.5
C9—C8—H8	119.7	S1—C26—H26B	109.5
C7—C8—H8	119.7	H26A—C26—H26B	109.5
C10—C9—C8	119.8 (3)	S1—C26—H26C	109.5
C10—C9—H9	120.1	H26A—C26—H26C	109.5
C8—C9—H9	120.1	H26B—C26—H26C	109.5
C11—C10—C9	119.9 (3)		
			
C1—Sn1—O1—Sn2^i^	90.30 (14)	O2^i^—Sn1—C7—C8	3.9 (2)
C7—Sn1—O1—Sn2^i^	−80.16 (12)	Cl1—Sn1—C7—C8	168.3 (2)
O2^i^—Sn1—O1—Sn2^i^	7.14 (8)	C12—C7—C8—C9	−0.3 (5)
Cl1—Sn1—O1—Sn2^i^	−177.90 (9)	Sn1—C7—C8—C9	179.4 (3)
C1—Sn1—O1—Sn2	−99.31 (19)	C7—C8—C9—C10	−0.9 (5)
C7—Sn1—O1—Sn2	90.23 (19)	C8—C9—C10—C11	1.6 (6)
O2^i^—Sn1—O1—Sn2	177.53 (19)	C9—C10—C11—C12	−1.0 (6)
Cl1—Sn1—O1—Sn2	−7.51 (16)	C8—C7—C12—C11	0.9 (5)
O1^i^—Sn2—O1—Sn1	−170.6 (2)	Sn1—C7—C12—C11	−178.8 (3)
C13—Sn2—O1—Sn1	−50.03 (19)	C10—C11—C12—C7	−0.3 (6)
C19—Sn2—O1—Sn1	76.80 (19)	O1^i^—Sn2—C13—C14	53.5 (3)
O2—Sn2—O1—Sn1	−168.40 (12)	C19—Sn2—C13—C14	−129.8 (2)
O1^i^—Sn2—O1—Sn2^i^	0.0	O1—Sn2—C13—C14	−23.1 (3)
C13—Sn2—O1—Sn2^i^	120.60 (10)	O2—Sn2—C13—C14	129.2 (2)
C19—Sn2—O1—Sn2^i^	−112.57 (10)	O1^i^—Sn2—C13—C18	−130.2 (2)
O2—Sn2—O1—Sn2^i^	2.2 (2)	C19—Sn2—C13—C18	46.5 (3)
O1^i^—Sn2—O2—Sn1^i^	−6.35 (7)	O1—Sn2—C13—C18	153.2 (2)
C13—Sn2—O2—Sn1^i^	−127.59 (9)	O2—Sn2—C13—C18	−54.5 (2)
C19—Sn2—O2—Sn1^i^	106.98 (10)	C18—C13—C14—C15	0.2 (5)
O1—Sn2—O2—Sn1^i^	−8.58 (18)	Sn2—C13—C14—C15	176.5 (3)
O1—Sn1—C1—C2	60.3 (3)	C13—C14—C15—C16	−0.3 (6)
C7—Sn1—C1—C2	−129.9 (2)	C14—C15—C16—C17	0.6 (6)
O2^i^—Sn1—C1—C2	133.3 (2)	C15—C16—C17—C18	−0.8 (6)
Cl1—Sn1—C1—C2	−28.1 (2)	C14—C13—C18—C17	−0.4 (5)
O1—Sn1—C1—C6	−127.6 (2)	Sn2—C13—C18—C17	−176.9 (3)
C7—Sn1—C1—C6	42.3 (3)	C16—C17—C18—C13	0.7 (5)
O2^i^—Sn1—C1—C6	−54.5 (2)	O1^i^—Sn2—C19—C24	−70.8 (3)
Cl1—Sn1—C1—C6	144.0 (2)	C13—Sn2—C19—C24	112.2 (3)
C6—C1—C2—C3	0.1 (5)	O1—Sn2—C19—C24	5.2 (3)
Sn1—C1—C2—C3	172.2 (3)	O2—Sn2—C19—C24	−146.2 (2)
C1—C2—C3—C4	−1.4 (6)	O1^i^—Sn2—C19—C20	112.1 (2)
C2—C3—C4—C5	1.9 (6)	C13—Sn2—C19—C20	−64.8 (3)
C3—C4—C5—C6	−1.0 (6)	O1—Sn2—C19—C20	−171.9 (2)
C2—C1—C6—C5	0.8 (5)	O2—Sn2—C19—C20	36.8 (2)
Sn1—C1—C6—C5	−171.6 (3)	C24—C19—C20—C21	0.1 (5)
C4—C5—C6—C1	−0.4 (6)	Sn2—C19—C20—C21	177.3 (3)
O1—Sn1—C7—C12	−102.1 (3)	C19—C20—C21—C22	0.3 (6)
C1—Sn1—C7—C12	86.9 (3)	C20—C21—C22—C23	−0.6 (6)
O2^i^—Sn1—C7—C12	−176.4 (3)	C21—C22—C23—C24	0.5 (7)
Cl1—Sn1—C7—C12	−12.0 (3)	C20—C19—C24—C23	−0.2 (5)
O1—Sn1—C7—C8	78.2 (3)	Sn2—C19—C24—C23	−177.3 (3)
C1—Sn1—C7—C8	−92.8 (3)	C22—C23—C24—C19	−0.1 (6)

Symmetry code: (i) −*x*+2, −*y*, −*z*+2.

### Hydrogen-bond geometry (Å, º)

**Table:**

*D*—H···*A*	*D*—H	H···*A*	*D*···*A*	*D*—H···*A*
O2—H2*A*···O3^ii^	0.82 (2)	2.04 (2)	2.851 (3)	166 (4)

Symmetry code: (ii) *x*+1, *y*, *z*+1.
